# Acute Oral Toxicity of Methanolic Seed Extract of *Cassia fistula* in Mice

**DOI:** 10.3390/molecules16065268

**Published:** 2011-06-23

**Authors:** Subramanion L. Jothy, Zuraini Zakaria, Yeng Chen, Yee Ling Lau, Lachimanan Yoga Latha, Sreenivasan Sasidharan

**Affiliations:** 1 Biological Program, School of Distance Education, Universiti Sains Malaysia, Minden 11800, Penang, Malaysia; 2 Institute for Research in Molecular Medicine (INFORMM), Universiti Sains Malaysia, 11800, Pulau Pinang, Malaysia; Email: srisasidharan@yahoo.com (S.S.); 3 Department of Parasitology, Faculty of Medicine, University of Malaya, 50603 Kuala Lumpur, Malaysia; Email: lauyeeling@um.edu.my

**Keywords:** *Cassia fistula*, methanol extract, acute oral toxicity, histology, hematology

## Abstract

*Background and objective*: *Cassia fistula* is widely used in traditional medicine to treat various types of ailments. The evaluation of toxic properties of *C. fistula* is crucial when considering public health protection because exposure to plant extracts can result in undesirable effects on consumers. Hence, in this study the acute oral toxicity of *C. fistula* seeds extract was investigated in mice. *Results*: Oral administration of crude extract at the highest dose of 5000 mg/kg resulted in no mortalities or evidence of adverse effects, implying that *C. fistula* in nontoxic. Throughout 14 days of the treatment no changes in behavioural pattern, clinical sign and body weight of mice in both control and treatment groups. Also there were no any significant elevations observed in the biochemical analysis of the blood serum. Further, histopathological examination revealed normal architecture and no significant adverse effects observed on the kidney, heart, liver, lung and spleen. *Conclusions*: Overall, the results suggest that, the oral administration of *C. fistula* methanolic seeds extract did not produce any significant toxic effect in mice. Hence, the extract can be utilized for pharmaceutical formulations.

## 1. Introduction

Traditional and alternative medicine is extensively practiced in the prevention, diagnosis, and treatment of various illnesses. It has attracted increasing public attention over the past 20 years as this type of medicine is easily accessible in some regions [1]. Plant-derived foods, particularly vegetables and fruits, are generally considered to be highly beneficial components of the human diet. They contribute great importance in daily life by providing wide range of nutrients, vitamins and other compounds which widen the therapeutic arsenal. In general, natural products play a dominant role in the development of novel drug leads for the treatment and prevention of diseases [2].

*Cassiafistula* L., (Caesalpinioideae), a semi-wild Indian Labernum also known as the golden shower is distributed in various regions including Asia, South Africa, China, West Indies and Brazil. *C. fistula* exhibits significant antifungal activity and also used in treatment of some diseases as a broad-spectrum antifungal agent [3]. According to the Indian literature, this plant has been described for its pharmacopeia uses. The whole plant is used to treat diarrhea and particularly the seeds, flower and fruits are used to treat skin diseases, fever, abdominal pain and leprosy [4]. The root possess astringent, tonic, febrifugal, and purgative properties and therefore, is useful against cardiac disorders, biliousness, rheumatic condition, haemorrhages, wounds, ulcers and boils, tubercular glands and various skin diseases [5,6]. Meanwhile, the flowers are eaten raw and it possess astringent, purgative, febrifugal and wound healing properties and a decoction of the flowers is given for stomach troubles. The pulp is a safe purgative due to the wax aloin, so it’s recommended for children and pregnant women [7]. It is given in disorders of liver, and in biliousness, and used as a tonic also applied in gout and rheumatism [8,9]. Apart from that, the pulp is used as an antipyretic and it is a remedy for malaria and blackwater fever [10]. It is also utilized in cases of blood-poisoning, anthrax and dysentery, and given in leprosy and diabetes and for the removal of abdominal obstructions. Meanwhile, the seeds are slightly sweet and possess laxative, carminative, cooling and anti-pyretic properties and they are given in cases of constipation [11]. Moreover, botanicals are enjoying widespread use of plants for treatment of several ailments, but still little known about their toxicity and safety issue which are always a concern. Investigations on functional plants provide evidence for the presence of substances that are offer potential human health benefits. However, it should be a vital requirement to determine the toxic effects of some of the substances contained in the plants [12].

Toxicity is an expression of being poisonous, indicating the state of adverse effects led by the interaction between toxicants and cells. This interaction may vary depending on the chemical properties of the toxicants and the cell membrane, as it may occur on the cell surface, within the cell body, or in the tissues beneath as well as at the extracellular matrix. The toxic effects may take place prior to the binding of the toxicants to the vital organs such as liver and kidneys. Hence, evaluation of toxic properties of a substance is crucial when considering for public health protection because exposure to chemicals can be hazardous and results to adverse effects on human being. In practice, the evaluation typically includes acute, sub-chronic, chronic, carcinogenic and reproductive effects [13].

The present study aims to determine the toxicity of methanolic seeds extract of *C. fistula* using an acute oral toxicity test in animal models [14]. The acute oral toxicity testing was carried out on both sexes of animals under the Organization for Economic Cooperation and Development (OECD) guidelines [15].

## 2. Results and Discussion

### 2.1. General Sign and Behavioural Analysis

The toxic effect of methanolic seeds extract of *C. fistula* on the appearance and the general behavioural pattern of mice are shown in Table 1 and Table 2 respectively. No toxic symptoms or mortality were observed in any animals, which lived up to 14 days after the administration of methanol seeds extract at single dose level of 5000 mg/kg body weight. The behavioural patterns of animals were observed first 6 h and followed by 14 h after the administration and the animals in both vehicle-treated and extract-treated groups were normal and did not display significant changes in behavior, skin effects, breathing, impairment in food intake and water consumption, postural abnormalities and hair loss. In the treated group in first 6 hrs rapid heartbeat was observed after the administration, but it then become normal and this may due to the stress of receiving the oral administration of the extract.

**Table 1:** Potential toxic effects of the crude extracts of *C. fistula* seeds in mice.

Control ^a^	Crude extract ^b^	Control ^a^	Crude extract ^b^
0/3 ^c^	0/3	0/3	0/3

^a^ Control groups (treatment without crude extract); ^b^ test groups (treatment with 5000 mg/kg crude extract); ^c^ Number of dead mice/ number of mice used.

**Table 2:** General appearance and behavioral observations for control and treated groups.

Observation	Control group		Test group	
	6 h	14 h	6 h	14 h
Skin and fur	Normal	Normal	Normal	Normal
Eyes	Normal	Normal	Normal	Normal
Mucous membrane	Normal	Normal	Normal	Normal
Behavioural patterns	Normal	Normal	Rapid heart beat	Normal
Salivation	Normal	Normal	Normal	Normal
Lethargy	Normal	Normal	Normal	Normal
Sleep	Normal	Normal	Normal	Normal
Diarrhea	Normal	Normal	Normal	Normal
Coma	N.O.^a^	N.O.	N.O.	N.O.
Tremors	N.O.	N.O.	N.O.	N.O.

^a^ Not Observed.

### 2.2. Organ and Body Weight Statistical Analysis

**Figure 1:**
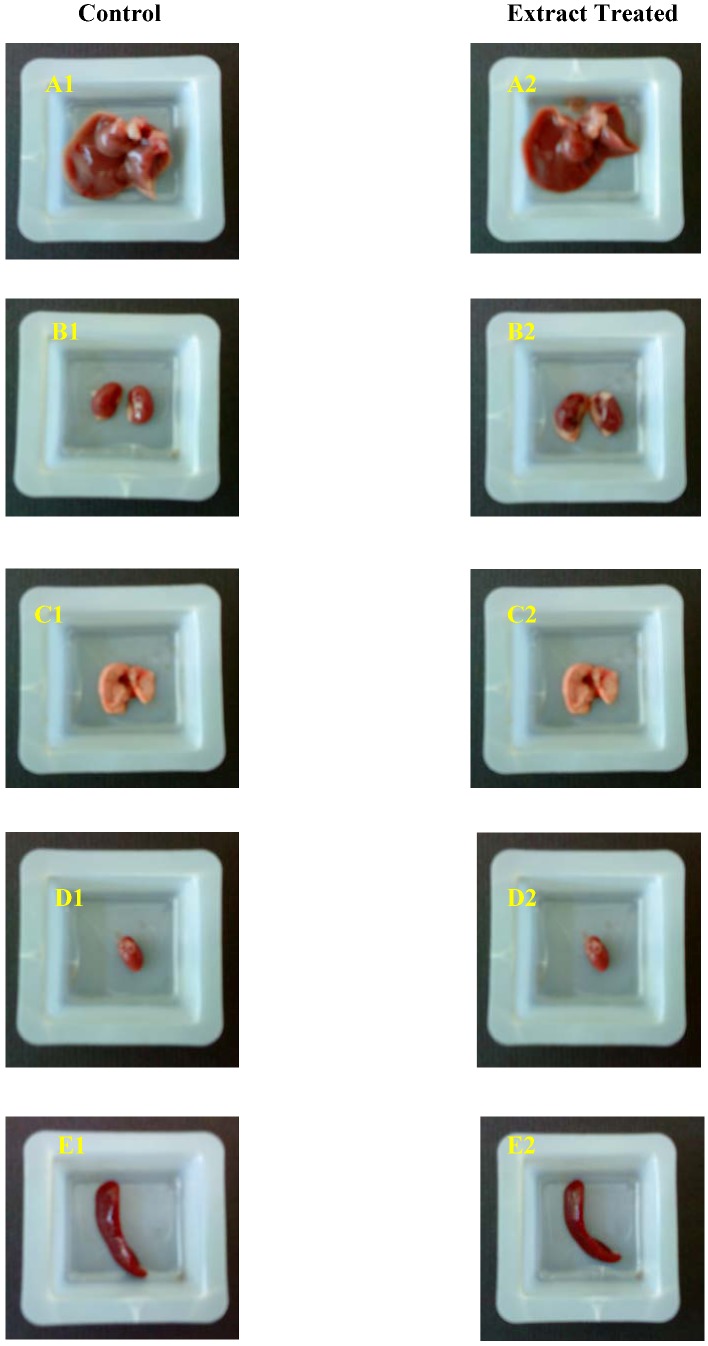
Gross observation of systemic organs: liver (A1 and A2), kidney (B1 and B2), lung (C1 and C2), heart (D1 and D2) and spleen (E1 and E2) from control and extract treated mice.

The gross observations of systemic organs from control and extract treated mice are shown in Figure 1. 

The body weight as well as weights of vital organs of the animals were calculated and are recorded in Table 3. There were no significant changes in body weight. However, the weight of the principal organs showed a significant increment. All animals exhibited a normal increment in body weight without drastic difference between both control and treated groups. Table 3 shows the effect of extract on principal organ weights relative to body weight. There were no significant differences in the changes of each weight. The results revealed that, the essential organs such as kidney, liver, heart, lung and spleen were not adversely affected throughout the treatment. The absolute and relative organ weight of mice between extract treated and control groups shown statistically significant differences (P < 0.05). Extract treated mice showed increased organ weight. 

**Table 3:** Effect of *C. fistula* seeds crude extract on organ-to-body weight index (%) in mice.

Organs	Organ body weight index	
	Treatment	Control
Kidney	1.46 ± 0.20 ^*^	0.94 ± 0.27
Heart	1.03 ± 0.27 ^*^	0.40 ± 0.02
Liver	9.27 ± 0.70 ^*^	4.93 ± 0.50
Lung	1.62 ± 0.53 ^*^	0.79 ± 0.03
Spleen	1.77 ± 0.49 ^*^	0.79 ± 0.38
Body Weight (g)	22.7 ± 0.79	25.1 ± 1.00

Organ body index = (organ weight × 100)/body weight; crude extract of *C. fistula* seeds was administered to mice at a dose of 5000 mg/kg; values are mean ± SD (n = 3) at 5% level of significance (* = P < 0.05).

### 2.3. Histopathology Analysis

**Figure 2:**
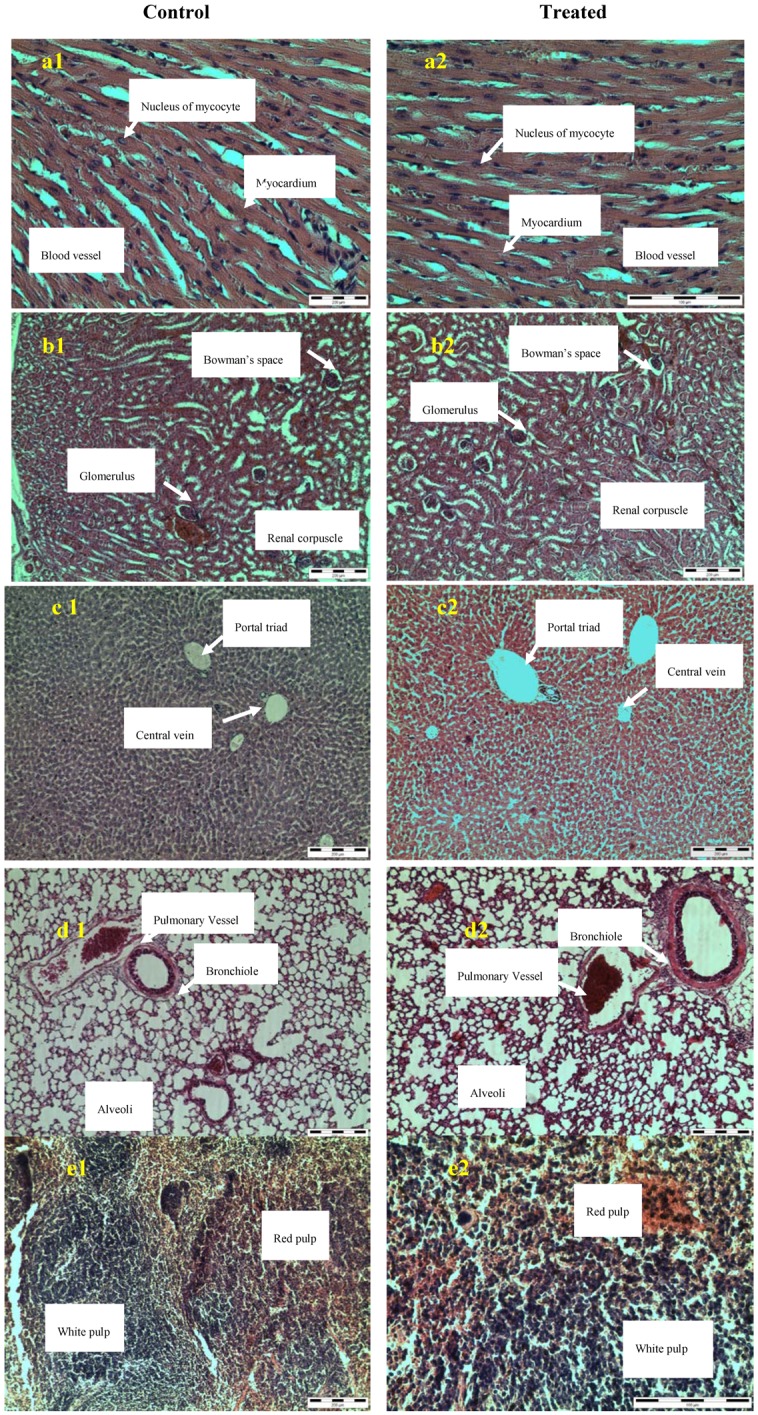
Histological examination of heart (**a**), kidney (**b**), liver (**c**), lung (**d**) and spleen (**d**).

Macroscopic examination of the organs of the animals treated with extract showed no changes in color compared to control. Autopsy at the end of the experiment period revealed no apparent changes in the liver, kidney, lungs, heart and spleen organs from both control and treated mice in the histopathology analysis. The microscopic structures of the organs depicted through Figure 2 shows unnoticeable differences between the control and test groups. The microscopic examination revealed that, all the organs from the extract treated mice did not show any alteration in cell structure or any unfavorable effects when viewed under the light microscope using multiple magnification power. The structure or coordination of cells in extract treated organs more or less similar compared with the control organs.

### 2.4. Biochemical Analysis

Table 4 shows the changes of biochemical parameters in the serum of female mice induced by *C. fistula* seed extract. In the female mice, there are no significant changes for the serum levels of total bilirubin levels (TBIL), alkaline phosphatase (ALP), alanine aminotransferase (ALT) and aspartate aminotransferase (AST) (*P* > 0.05) after oral administration of *C. fistula* seed extract.

**Table 4:** Changes of biochemical parameters in the serum of mice induced by *C. fistula seed* extract.

Groups	TBIL (mmol/L)	ALT (U/L)	AST (U/L)	ALP (U/L)
**Control**	0.8 ± 0.2	15.5 ± 5.4	79.4 ± 24.2	78.3 ± 18.9
**Treated**	0.9 ± 0.5	16.3 ± 4.3	78.7 ± 22.6	77.9 ± 19.7

### 2.5. Discussion

Phytotheraputic products from medicinal plants have become universally popular in primary healthcare, particularly in developing countries, and some have been mistakenly regarded as safe just because they are a natural source. Nevertheless, these bioactive products from medicinal plants are presumed to be safe without any compromising health effect, and thus widely used as self medication [16]. However, there is a lack of proven scientific studies on the toxicity and adverse effect of these remedies. Therefore, further acute oral toxicity study is vitally needed not only to identify the range of doses that could be used subsequently, but also to reveal the possible clinical signs elicited by the substances under investigation. It is also a useful parameter to investigating the therapeutic index of drugs and xenobiotics [17].

In general *in vivo* toxicity study is the toxicological analysis of many medicinal plants and its potency to evaluate qualitatively and quantitatively by histopathology and oral acute toxicity studies. Oral acute toxicity testing in mice could be used to evaluate natural remedies for different pharmacological activities, taking into account the basic premise that pharmacology is simply toxicology at a lower dose [18]. A toxic substance might elicit interesting pharmacological effects at a lower non-toxic dose. Toxicity results from animals will be crucial in definitively judging the safety of medicinal plants if they are found to have sufficient potential for development into pharmacological products [19]. As use of medicinal plants increases, experimental screening of the toxicity of these plants is crucial to assure the safety and effectiveness of those natural sources. However, acute toxicity studies do not detect effects on vital functions like the cardiovascular, central nervous, and respiratory systems which are not usually assessed during the study and these should be evaluated prior to human exposure [20]. Moreover, acute toxicity is mainly to obtain an appropriate dose for long-term toxicity tests and to find out the affected organs at the end of the treatment. The previous study on preliminary toxicity analysis of *C. fistula* seeds extract by using brine shrimp lethality test have documented the seeds extract are not toxic and shows that, the extract can further explored for the development of natural product based pharmaceutical products [21]. Hence, the present study was particularly designed to further investigate toxicity of methanolic seeds extract of *C. fistula* by using acute oral toxicity analysis. 

In this oral acute toxicity study, the Swiss albino mice were employed to observe the toxicity effects of the methanol crude extract of *C. fistula* seeds. The route of administration depends on the dosage form in which the compound is available. Based on historical research, the oral route administration is the most convenient and commonly used one when studying acute toxicity. The absorption might be slow, but this method costs less and is painless to the animals. Since the crude extract is administered orally, the animals should be fasted before taking the dose because food and other chemicals in the digestive tracts may affect the reaction(s) of the compound. Although there is a problem regarding extrapolating animals’ data to humans, a study has shown that mice give better prediction for human acute lethal dose compared to rats [22]. All the procedures were performed based on the appropriate OECD guideline [15].

In this study, the mice in the control and treated groups were administrated with vehicles and crude extract, respectively. The mice were monitored daily until day fourteen for any toxic signs and mortality. The clinical symptom is one of the major important observations to indicate the toxicity effects on organs in the treated groups [23]. During the 14 days of period acute toxicity evaluation, mice which are orally administrated with methanolic seeds extract at single dose 5000 mg/kg showed no overt signs of distress, and there were no observable symptoms of either toxicity nor deaths. All of the mice gained weight and displayed no significant changes in behavior. Apart from that, the physical appearance features such as skin, fur and eyes were found to be normal and whilst the body weight of the mice showed as increase (Table 2 and Table 3), this indicates that the administration of the crude extract has negligible level of toxicity on the growth of the animals. Furthermore, determination of food intake and water consumption is important in the study of safety of a product with therapeutic purpose, as proper intake of nutrients is essential to the physiological status of the animal and to the accomplishment of the proper response to the drugs tested [24,25]. In this study, the food intake and water consumption also was not affected by the administration of methanolic seeds extract *of C. fistula* and it did not induce appetite suppression and had no deleterious effects. Thus, this indicates there was no disturbance in carbohydrate, protein or fat metabolism [26]. 

Generally, the alterations of body weight gain and internal organ weights of mice would reflect the toxicity after exposure to the toxic substances [27]. The body weight changes are indicators of adverse effects of drugs and chemicals and it will be significant if the body weight loss occurred is more than 10% from the initial weight [28,29]. Organ weight also is an important index of physiological and pathological status in animals. The relative organ weight is fundamental to diagnose whether the organ was exposed to the injury or not. The heart, liver, kidney, spleen and lungs are the primary organs affected by metabolic reaction caused by toxicant [30]. The gross observation of systemic organs of both control and treated groups are shown in Figure 1. There is no changes were observed in gross observation of systemic organs of both control and treated groups. In this study, the relative and absolute of organs weight in both control and treated groups was increased significantly which shows that the extract nurtures the organs (Table 3). Differently, then body weight gain was same in both control and treated groups the difference were not statistically significant. The, administration of methanolic seeds extract did not show any adverse affect on organs weight of all important organs. Hence, it can be suggested that, *C. fistula* seeds extract is virtually nontoxic. 

This study reckoned that *C. fistula* seeds extracts do not cause acute toxicity effects and an LD_50_ value greater than 5000 mg/kg. In principle, the limit test method is not intended for determining a precise LD_50_ value, but it serves as a suggestion for classifying the crude extract based on the expectation at which dose level the animals are expected to survive [31]. According to the chemical labeling and classification of acute systemic toxicity recommended by OECD, the crude extract of *C. fistula* seeds was assigned class 5 status (LD_50_ > 5000 mg/kg) which was the lowest toxicity class. According to the study by Kennedy *et al*. [32] substances with LD_50 _values higher than 5000 mg/kg by oral route are regarded as being safe or practically non-toxic. Meanwhile, a study done by Ilavarasan *et al*. [33] using *Cassia fistula* methanolic bark extract revealed that extract did not cause any mortality up to 2000 mg/kg and was thus considered as safe. Similar results were found for a single dose at 2000 mg/kg oral administration of *C. spectabilis* leaf extracts that was shown to be non-toxic to the tested mice [34].

Apart from that, histological analysis was done to further confirm the alteration in cell structure of the organs. The histological examination is the golden standard for evaluating treatment related pathological changes in tissues and organs [35]. In the present study, histopathological evaluation of acute oral ingestion of *C. fistula* seeds indicated that the extract did not adversely affect the morphology of mice organs. This agrees with the results of biochemical analysis, and oral administration of 5000 mg/kg for 14 days was well tolerated by the treated mice. In general, the histopathology analysis collaborated with the results of body weight and organ weight. The seeds extract of *C. fistula* did not cause toxicity towards the organs as there was no structural damage to the tested organs of liver, kidney, lungs, and spleen of the mice. The liver is the main target organ of acute toxicity where exposed to the foreign substances being absorbed in intestines and metabolized to other compounds which may or may not be hepatotoxic to the mice [36]. In this study, the liver histology revealed evidence determines normal hepatocytes and did not cause any alteration to the structure of the liver cells between the controls and treated (Figure 2c). In contrast, the histological examination study conducted by Harizal *et al*. [37] using *Mitragyna speciosa* extract revealed less severe morphological changes in liver of mice treated with extract at dose level 100 and 500 mg/kg. Meanwhile, the other study by Salawu *et al*. [38] using *Crossopteryx febrifuga* saw inflammatory changes histologically in the liver by infiltration of lymphocytes at portal and central of rat treated with at dose level 500 and 1000 mg/kg and this shows that, the extract exerted deleterious effects on the liver. The liver is capable of regenerating damaged tissue, hence the liver function may not be impaired early following an insult from a toxicant [38]. Apart from that, the acute toxicity study conducted on *C. fistula* pod extract and histological examination of the organs of rat treated with extract at a dose of 1000 mg/kg revealed that there was no potential toxicity or damage to the cell structure of liver, kidney and testes. Also there was no necrosis, inflammatory reaction, fibrosis or local fatty degeneration observed in liver and the arrangement of cell structure almost similar to the organs of rats in control groups [39]. The histological features liver from this study are displayed in Figure 2c and marked with central vein and portal triad. The morphology of liver cell in both control and treated groups are normal and no structural damages were observed. 

The kidney micrograph displayed in Figure 1b shows that no adverse effects were observed in both groups and the glomeruli and capsules appeared normal and the Bowman’s space are also marked clearly. In contrast, the study conducted by Alade *et al*. [40] revealed the histology of kidney observed with focal proximal tubular epithelial necrosis, meanwhile there was variation in the lung between the control and treated with rat *B. monandra* leaf extract at dose 4 g/kg. Apart from that, the study conducted by Akanmu *et al*. [39] on *C. fistula* pods extract revealed that there were slight changes in the histology of kidney from the rat treated with extract at dose 1000 mg/kg where some of the glomeruli and the proximal tubules was observed to widen without any injury compared to the control. Furthermore, in this study the microscopic inspection of the lung and heart (Figures 2 a,d) of the mice treated with extract did not indicate any changes in the organs compared to control mice. Meanwhile, histology of the spleen (Figure 2e) of mice in both control and treated groups are well labeled with white pulp and red pulp.

The hematopoietic system is very sensitive to toxic compounds and serves as an important index of the physiological and pathological status in both animals and humans [41]. After 14 days of treatment with *C. fistula* seeds extract there was no changes in the hematological parameters between the control and treatment groups. This indicates that there were no significant changes of serum levels of TBIL, ALP, ALT and AST hence, verifying the non toxic nature of *C. fistula* seed extract. This finding also supports the usage of these seeds in traditional medicine by the traditional healers. However, the normal range of this parameter can be altered by the intake of toxic plants [42] which was not observed in this study. 

## 3. Experimental

### 3.1. Plant Materials

Fresh pods of *Cassia fistula* were collected from various areas in Universiti Sains Malaysia, Penang in November 2010 and authenticated by the botanist of the School of Biological Sciences at Universiti Sains Malaysia where the herbarium sample was deposited. The sun-dried pods were cut open and the seeds removed from the pods. The seeds were then washed thoroughly and rinsed with tap water and dried in oven at 60 °C for three to four days. Then the dried seeds were homogenized to a fine powder and stored in airtight bottles. 

### 3.2. Preparation of Crude Extracts

The seed sample was sequentially extracted with methanol by adding approximately 100 g of the dried sample (in fine powder form) to 400 mL methanol. The extraction was carried out at room temperature by soaking for 7 days with intermittent stirring during the first day. The extracts were filtered through clean muslin cloth and the extraction process was repeated again for a second time by adding another 400 mL of methanol to the sample residue. The filtrate from each extraction was combined and concentrated under vacuum on a rotary evaporator (Buchi, Switzerland) at 40 °C to 50 °C in order evaporate the excess methanol solvent and until a dark green methanol extract was produced. The concentrated extract was poured into glass Petri plates and brought to dryness at 60 °C in the oven until a paste-like mass was obtained. Then paste form extract was sealed in Petri plates and stored at room temperature (RT). The crude extract was prepared by diluting the paste in methanol and storing in air-tight bottles at 4 °C for further studies.

### 3.3. Acute Oral Toxicity Study

#### 3.3.1. Target Animal

The experiment was conducted on 12 healthy Swiss albino mice (males and females) weighing 25 g to 35 g and aged 8 to 10 weeks obtained from the Animal House, Universiti Sains Malaysia Penang. The mice were distributed into two groups. The experimental procedures relating to the animals were authorized Universiti Sains Malaysia Ethical committee (USM/ Animal Ethics Approval/ 2010/ (59) (262)) before starting the study and were conducted under the internationally accepted principles for laboratory animal use and care. 

#### 3.3.2. Acute Toxicity Assay

The mice were housed in cages and randomly selected ones were marked on the tail for individual identification. All mice were maintained on a 12-h light/dark cycle and located at room temperature approximately 23 °C with constant humidity. They were allowed to acclimatize to laboratory conditions for a week before starting the experiment. Drinking water and food were provided *ad libitum* throughout the experiment, except for the short fasting period where the drinking water was still in free access but no food supply was provided 12 h prior to treatment. The acute oral toxicity methanolic seed extract of *C. fistula* was evaluated in mice according to the procedures outlined by the Organization for Economic Co-operation and Development (OECD) [15]. A single high dose of 5,000 mg/kg of crude extract was administered to both three male mice and three female mice in the treatment groups by the oral route. The crude extract was suspended in a vehicle (distilled water). Following the fasting period, body weight of the mice were determined and the dose was calculated in reference to the body weight as the volume of the extracts solution given to the mice is 10 mL/kg. Another three male mice and three female mice were allotted distilled water and were regarded as the control groups. Food was provided to the mice approximately an hour after treatment. The mice were observed in detail for any indications of toxicity effect within the first six hours after the treatment period, and daily further for a period of 14 days. Surviving animals were weighed and visual observations for mortality, behavioral pattern, changes in physical appearance, injury, pain and signs of illness were conducted daily during the period.

### 3.4. Histopathological Analysis

#### 3.4.1. Organs and Body Weight Statistical Analysis

Finishing the 14 days period, all the mice were sacrificed. Vital organs such as heart, kidneys, liver, lung and spleen were isolated and examined for any lesions. All of the individual organs were weighed and their features were compared between both treated and control groups. 

#### 3.4.2. Histopathology of Heart, Kidneys, Liver, Lung and Spleen

All the vital organs isolated from each individual were fixed in 10% buffered formalin, routinely processed and embedded in paraffin wax. Paraffin sections (5 µm) were cut on glass slides and stained with haematoxylin and eosin. The slides were examined under a light microscope and the magnified images of the tissues structure were captured for further study [43].

#### 3.4.3. Blood Biomarker Assay

After 14 days of treatment with *C. fistula* seeds extract the mice blood were further evaluate for biochemical analysis. In the present study, the liver function was evaluated with serum levels of TBIL, ALP, ALT and AST.

#### 3.4.4. Statistical analysis

Statistical analysis involved use of the Statistical Package for Social Sciences (SPSS). Data are given as the Mean ± SD; statistics were performed using *t*-tests and *p* values less than 5% were considered statistically significant (*p* < 0.05).

## 4. Conclusions

The present results show that methanol seed extract of *C. fistula* does not cause any apparent *in vivo* toxicity in an animal model. No death or signs of toxicity were observed in mice treated with extract at dose 5000 mg/kg thus establishing its safety in use. The histology examination revealed no changes in the architecture of the internal organs mice in both control and treated groups. Hence, *C. fistula* can be used as a medicinal agent in known dosages, especially in rural communities where conventional drugs are unaffordable because of their high cost. A detailed experimental analysis of its chronic toxicity is essential for further support of this drug.
